# The influence of the Nutri-Score on the perceived healthiness of foods labelled with a nutrition claim of sugar

**DOI:** 10.1371/journal.pone.0272220

**Published:** 2022-08-17

**Authors:** Kristin Jürkenbeck, Clara Mehlhose, Anke Zühlsdorf

**Affiliations:** Department of Agricultural Economics and Rural Development, Marketing of Food and Agricultural Products, University of Goettingen, Goettingen, Germany; The University of Hong Kong, HONG KONG

## Abstract

High sugar intake in humans is associated with the development of overweight and other diet-related diseases. The World Health Organization and other health organizations recommend limiting the sugar intake to 10% of the total energy intake. There have been different approaches of front-of-pack labelling to reduce the amount of sugar in food products. Companies use nutrition claims to advertise the sugar content (e.g., without added sugar, 30% less sugar). Such nutrition claims can lead to false assumptions about the healthiness of foods and can lead to health-halo effects. Nutrition claims make products appear healthier than they really are, the aspect advertised in the nutrition claim is transferred to the entire food product. As a result, food products can be perceived as healthy even though they are not. Recently, the Nutri-Score was introduced in an increasing number of countries throughout Europe to provide consumers with an overview of the overall nutritional quality of a product. This study analyzes if the Nutri-Score can help to prevent health-halo effects caused by nutrition claims on sugar. Therefore, an online survey consisting of a split-sample design with more than 1,000 respondents was assessed. The results show that, depending on the initial perceived healthiness of a product, the Nutri-Score is able to prevent health-halo effects caused by claims on sugar. Making the Nutri-Score mandatory when using nutrition claims would be one possible way to reduce misperceptions about unhealthy food and reduce health-halo effects caused by claims on sugar.

## Introduction

High sugar intake is associated with the development of overweight and other diet-related diseases, e.g., Type 2 diabetes and cardiovascular diseases in humans [1]. The World Health Organization suggests limiting intake of ‘free sugar’ to less than 10% of total energy intakes, with recommendations to further reduce down to 5% for additional benefits [2, 3]. Free sugar are all mono- and disaccharides excepts those that are naturally occurring. Added sugar consists of all sugars added to the food product by the manufacturer, cook or consumer and are included in free sugar. Total sugar includes mono- and disaccharides in food from any source—including naturally occurring sugar in fruits and vegetables [4].

In terms of nutrition policy, reducing the amount of added sugar in food is a key goal of many European countries [5]. In Germany, for example, the government has reached target agreements with the food industry under the National Reduction and Innovation Strategy to reduce the content of sugar, fat and salt in certain finished products by 2025. [6] Nine food industry associations have so far concluded process or target agreements with their member companies [7].

Additionally, the public is increasingly aware of the need to reduce the sugar intake, and a growing number of consumers are paying attention to foods that advertise a low sugar content [7–9].

Research shows that the food environment is underestimated in public and political debates, while individual control of action is overestimated [10]. According to a systematic review by Pitt et al. [11], three key determinants of store choice and purchasing behavior are availability, accessibility, and affordability. Availability and accessibility were also identified as important measures in research by Bivoltsis et al. [12]. The exposure to food (e.g., advertisement, portion sizes in restaurants) often directs consumer awareness towards food with unfavorable nutritional profiles [10]. The food environment needs to be modified and improved to maximize health-related outcomes [11].

In most countries, consumers can infer the nutritional quality of foods when shopping at the supermarket by looking at various indications on the packaging. First, the nutrition fact table on the back of the product, second, front-of-pack nutrition labels (e.g., Nutri-Score), and third, nutrition and health claims on the packaging. The nutrition fact table is mandatory on food products, while front-of-pack labels are voluntary in many countries. Only a minority of consumers use the nutrition fact table to get information about the nutritional quality of food items [13]. Reasons cited in research include inconspicuous placement on the back of packaging and problems interpreting the numerical information [14]. Instead, information on the front-of-pack, such as nutrition labels, is a more effective tool to draw attention to nutrition information [15]. More recently, the discussion is focused on interpretive summary indicator labels, which provide an interpretive aggregation of nutrition information summarizing the overall nutritional value of a food (e.g., Nutri-Score, Health Star Rating) [15, 16].

Nutrition and health claims highlight the beneficial nutritional character of a food item. In Europe, the European Commission Regulation 1924/2006 reports the allowed definitions for nutrition and health claims [17]. A health claim is ‘any claim which states, suggests or implies that a relationship exists between a food category, a food or one of its constituents and health’ [17]. In contrast, a nutrition claim is ‘any claim that states, suggests or implies that a food has particular beneficial nutritional properties due to the energy, nutrients or other substances it contains, contains in reduced or increased proportions or does not contain’ [17]. If nutrition claims are on the front-of-pack label, there is a risk that detailed information, such the nutrition fact table, is noticed even less [18, 19]. This means that consumers may pay more attention to nutrition claims than to the actual nutritional values of the food.

In addition, the number of food products containing a reduced (e.g., sugar) or higher (e.g., protein) level of specific ingredients is growing. The food industry is increasingly using nutrition claims related to the sugar content to market their product lines. These claims are known as sugar claims, and examples include ‘without added sugar’ and ‘sugar reduced’. Additionally, food companies are also using claims concerning the sweet taste of a product, e.g., less sweet, also known as ‘taste claims’ [20].

Such taste claims carry the risk that consumers associate them with a reduced sugar content. However, taste claims are only related to a taste description and do not provide any information about the sugar content, and thus conclusions about the nutritional quality of a product are not possible. Research shows that nutrition claims are effective in targeting health-oriented consumers [21]. This may lead to difficulties in evaluating the healthfulness of a food, as a single positive characteristic of a food item can influence the overall perception of a food product [22]. Bernstein et al. [23] found that products containing sugar claims are perceived to be healthier than the same products without such a sugar claim. This phenomenon is called halo effect [24] because the claim (single positive characteristic) changes the overall health perception of the product. As, in this research, the halo effect is related to health, it is called health-halo effect. The health-halo effect may be a problem when the nutritional quality of a food product does not match consumers’ perception and, in reality, the food is not healthier than comparable foods without such a claim. Past research highlights this risk of misperception for various products, e.g., protein bars, breakfast cereals, fruit snacks, fruit-based drinks, and ready-made meals [21, 25–27]. Moreover, 1/3 of less healthy products contain a nutrition or health claim which may mislead consumer perception of the nutritional quality [28]. A literature review found that food products containing a nutrition claim related to fat, sugar, or energy make them appear healthier and less tasty [29]. Moreover, warning labels can mitigate, but not eliminate, the influences of nutrition claims on consumer perceptions of product health [30].

Against this background, the first objective of this research was to find out if sugar and taste claims make foods appear healthier than they actually are. As the Nutri-Score (detailed explanation can be found in the method section) gives consumers an overview of the nutritional quality of food products [31] the second objective of this study was to assess how the Nutri-Score, in combination with nutrition claims about the sugar content and taste claims about the level of sweetness, influences consumers’ evaluation of the perceived healthiness of these products. To answer these two objectives, this research consists of a split-sample design to test whether the presence of the Nutri-Score can prevent consumers’ misperception of a food’s healthiness based on sugar and taste claims on the front-of-pack labels.

## Materials and methods

### Nutri-Score

The Nutri-Score is an interpretive front-of-pack label that shows the nutrient quality on a graded scale of five colors, from dark green to red, in combination with a letter (A to E). The Nutri-Score algorithm combines positive characteristics with negative characteristics to achieve a score between −15 (healthy) and +40 (unhealthy). The dark green A reflects the highest nutritional quality, while the red E stands for the lowest nutritional quality [32]. The Nutri-Score is a voluntary label system that is becoming increasingly important in the European market. For example, its use is supported by governments in France, Spain, Belgium, and Germany. However, at the time of the survey, the Nutri-Score was not yet officially recommended by the German government and the Nutri-Score was not widespread in the market.

Research has shown that the Nutri-Score is an intuitively understandable label compared to other labels [31, 33]. Moreover, the Nutri-Score has proven to be an effective tool for guiding and steering consumers toward more informed and healthier food purchase decisions [33, 34]. The classification of foods in the Nutri-Score is according to the German Food-Based Dietary Guidelines (FBDG) [35]. In a large-scale purchasing experiment (included labels: SENS, Nutri Repère, Nutri-Couleurs, and Nutri-Score) in French supermarkets it was the only label which improved the nutritional quality of the shopping basket of labelled products purchased, and it has a positive effect on the purchase of products with high nutritional quality, followed by a reduced effect on the purchase of products with medium and low nutritional quality [36].

### Ethic statement

Participants provided their electronic consent in the beginning of the online survey. They were able to withdraw from participation by just closing the survey or browser. No data can be identified and/or linked to individual participants and all participants were informed that data would be anonymously analyzed. Data collection was in full accordance with the strict guidelines to the German DSGVO. The survey is in accordance with the Association of German Professional Psychologists (BDP) and with the Ethical Principles of the German Psychological Society (DGP).

### Data collection and survey design

An online survey was conducted in Germany in October 2020. A professional online access panel provider (respondi) was responsible for data collection. Quota sampling was used with gender, age, education, income, and the region of residence as quota parameters to mimic the German population in these characteristics.

To ensure data quality, two quality check questions were included. If participants incorrectly answered the questions, they were directly excluded from the survey (n = 276). Additionally, respondents who answered too fast (below ½ of the average response time (n = 71)), or with stereotypical response behavior (e.g., straight-liners (n = 12)) were eliminated from the dataset. After this procedure, 1,103 valid respondents (n = 1,103) remained in the data set for analysis. The raw data file is available via the GRO.data repositorium (https://doi.org/10.25625/FEKUCY). GRO.data is managed by the University of Goettingen and publicly accessible. The sociodemographic sample parameters are shown in Table 1.

**Table 1:** Sample description.

Variable	Characteristics	Sample (%)	German Population^a^ (%)
**Gender**	Male	49.1	49.4
	Female	50.6	50.6
	Divers	0.3	-
**Age**	16–29	18.6	18.1
	30–49	29.3	29.5
	50+	52.1	52.4
**Education**	No qualification (yet)	21.7	21.8
	Apprenticeship	50.5	50.5
	Technical college degree	9.7	9.6
	University degree	18.1	18.8
**Region**	West Germany	82.9	80.5
	East Germany	17.1	19.5
**Income (in Euro)**	Up to 1,300	25.8	26.3
	1,300–2,599	39.9	39.6
	2,600–4,999	27.3	27.1
	5,000 +	7.0	6.5

^a^Source: Statistisches Bundesamt (2020) [37].

**Fig 1:**
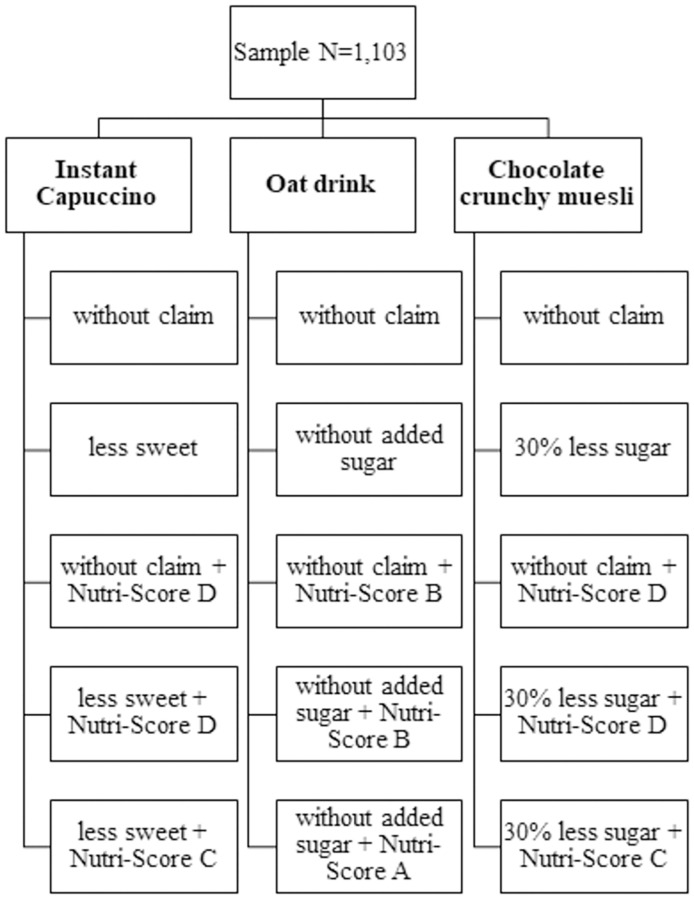
Overview of the three products and included claims. Note: A split sample design was used. ’Without claim’ means that this subsample had no claim on the product.

After having answered the sociodemographic questions, respondents had to rate items relating to their food shopping behavior on a Likert scale ranging from 1 = never to 7 = always. Participants were shown a picture of the Nutri-Score and asked if they knew its meaning. The scale ranged from 1 = I know for sure what this sign means to 5 = I have no idea what this sign could mean. In the survey, the meaning of the Nutri-Score was not explained in order to simulate a situation comparable to shopping for the product assessment. Three products consisting of different nutrition profiles (instant cappuccino, oat drink, and chocolate crunchy muesli) had to be evaluated regarding their health value on a Likert scale from 1 = very healthy to 10 = very unhealthy. The instant cappuccino and the chocolate crunchy muesli were chosen as product examples of a medium to poor Nutri-Score rating, while the oat drink represented products consisting of a positive Nutri-Score rating. The ratings for the products were based on real examples from the supermarket. Afterwards, respondents were randomly allocated to one of five claim variations per product example. The random assignments to the claims led to a split sample design of 3 products x 5 claim variations (Fig 1).

**Fig 2:**
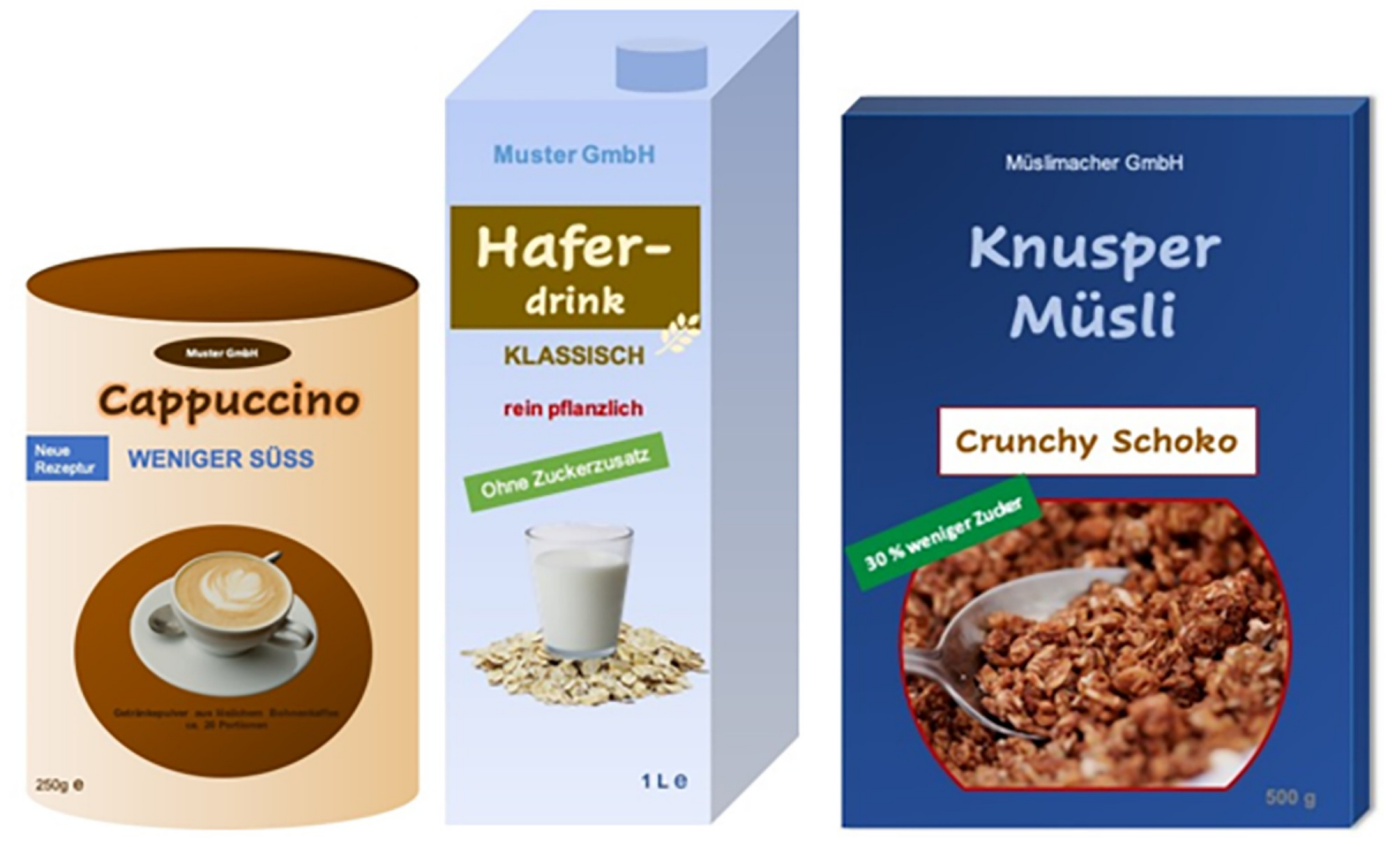
Product examples with the three claim variations: Instant cappuccino (less sweet), oat drink (without added sugar), chocolate crunchy muesli (30% less sugar).

**Fig 3:**
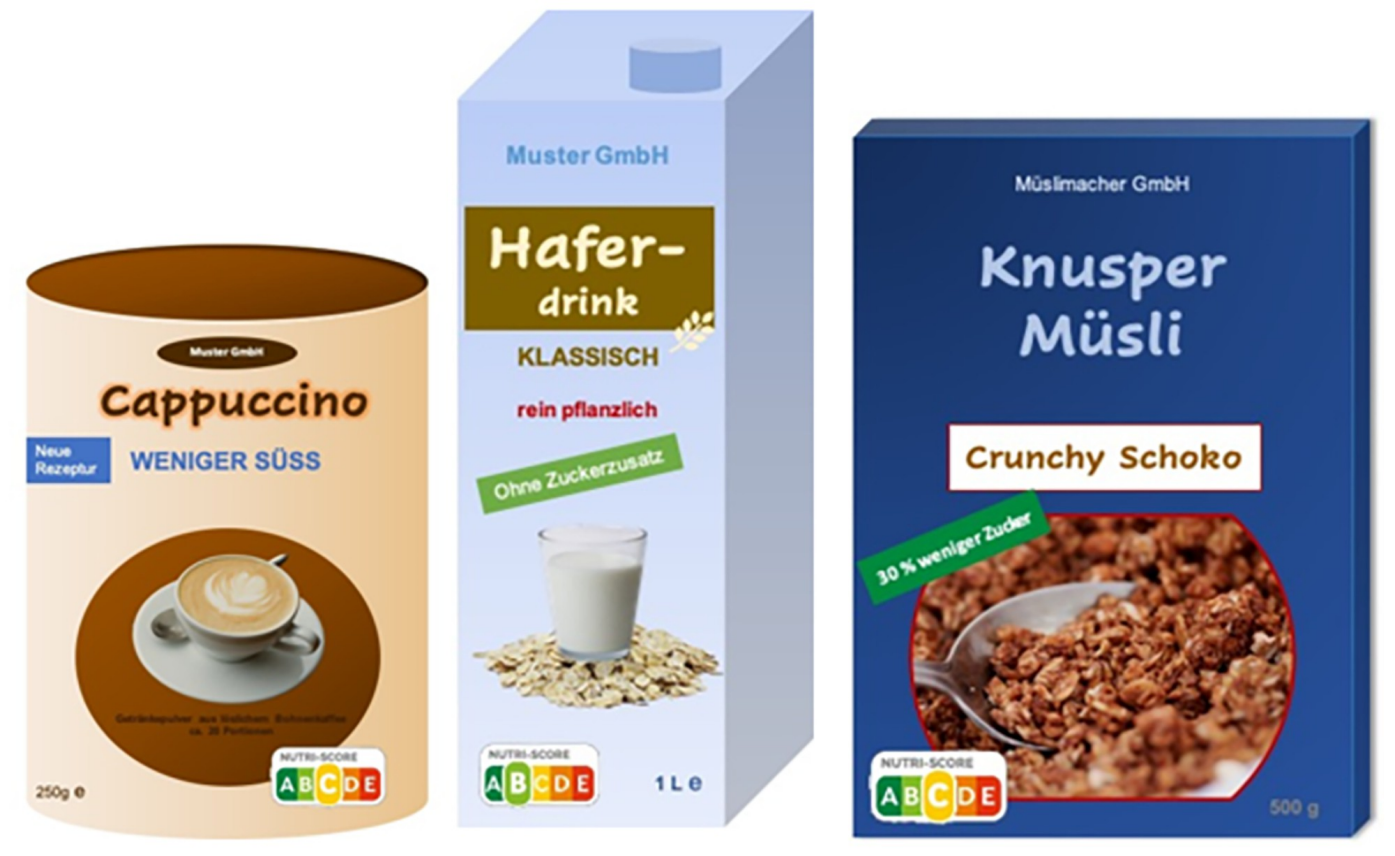
Product examples with the three sweet claim variations and the Nutri-Score.

Claims about the sugar content are very popular and widely used on products in Western Europe and the US [38, 39]. Therefore, the two claims ‘without added sugar’ and ‘30% less sugar’ are permitted nutrition claims established by the Commission Regulation Number 1924/2006 of the European Commission [17]. Claims that state that sugars have not been added, e.g., ‘without added sugar’, may only be made if the respective products do not contain mono- or disaccharides, or other ingredients used for their sweetening effect. If ingredients contain sugar by nature, this should be indicated; however, this is not mandatory. The claim ‘30% less sugar’ means that the sugar content of the food must be reduced by at least 30% compared to other foods of the same product category. The claim is only permissible if, in addition, the energy content (kcal) is equal to or lower than that of comparative products [17]. In contrast, the taste claim ‘less sweet’ is not defined by law. Additionally, based on the results of a principal component analysis of a preliminary study (which consisted of n = 1,312 respondents with the same quota parameters as described above) the claim ‘less sweet’ was categorized within the ‘reduction claims’, meaning that consumers judge them as such [40]. This shows the misleading character of such taste claims. Fig 2 shows examples of each product consisting of one taste or nutrition claim (weniger süss = less sweet, ohne Zuckerzusatz = without added sugar, and 30% weniger Zucker = 30% less sugar) and in Fig 3 with the corresponding Nutri-Score.

### Statistical analysis

The statistical analysis was undertaken by using the software SPSS 26. To find out if the different claim variations influence consumers’ perceived health evaluation of the products, analysis of variance, including Games-Howell or Turkey as post hoc tests, were used. Additionally, an analysis of variance was chosen to test if the Nutri-Score influences consumers’ perceived health evaluation when such claims are visualized on the products.

## Results

### Descriptive results

When purchasing food, consumers paid the highest attention to the ingredients (4.22), followed by the sugar content (4.13) and fat content (3.77) of a product. The results can be seen in Table 2.

**Table 2:** Importance of food characteristics in grocery shopping.

Item	Mean	SD
Ingredients	4.22	1.76
Sugar content	4.13	1.90
Fat content	3.77	1.80
Calorie content	3.73	1.85
Food additives	3.68	1.88
Vitamin content	3.36	1.71
Whole grain content	3.32	1.82
Carbohydrate content	3.32	1.83
Protein content	3.03	1.81
Salt content	2.96	1.74

Note: Question: When you buy food, you pay attention to different aspects. Do you personally pay attention to the following aspects when shopping, and do you read the information on the packaging? Scale from 1 = never to 7 = always, SD = standard deviation.

The meaning of the Nutri-Score was known by 32.8% of the respondents, while 37.5% could roughly imagine what the label means, and 29.6% had no idea what the label could mean. The health perception differed between both the product examples and between the product variants investigated. A rating of respondents was classified as healthy if the respondents rated it on the Likert scale with a value between 1–5, while a rating between 7–10 was classified as unhealthy. Of all the products, the chocolate crunchy muesli was considered the unhealthiest product. The instant cappuccino was rated somewhat more positively, and the oat drink had a clearly positive health image.

### Health evaluation of the products variations

The differences in health perception varied in the respective product variants and were not statistically significant in all cases. The health assessments for the various claim and label variants of a product also differed from one another. The neutral package of the instant cappuccino (meaning no claim or Nutri-Score on the packaging) was rated nutritionally rather unhealthy by almost 70% of the respondents. The claim ‘less sweet’ improved the health value by 11%, meaning that only 59% rated it as unhealthy. When only the Nutri-Score D was shown on the packaging, 82% of the respondents valued the instant cappuccino to be unhealthy. The effect of the Nutri-Score D, which was highlighted in light red, was stronger than the Nutri-Score C, which was yellow. In comparison, the oat drink without claim was rated as healthy by 84% of the respondents. It was the product example with the most positive health value without a claim or Nutri-Score influence. The health value of the oat drink did not increase when the claim ‘without added sugar’ was on the package. As this was a product which was perceived as healthy, the Nutri-Score only slightly increased the health evaluation. The chocolate crunchy muesli without claim was perceived as unhealthy by 78.9% of the respondents. The claim ‘30% less sugar’ increased the positive assessment as healthy by 16.9%. In combination with the Nutri-Score D, the positive assessment as healthy was diminished. When comparing the chocolate crunchy muesli and the instant cappuccino, the health assessment of the chocolate crunchy muesli was not reduced by the combination of ‘without claim and Nutri-Score D’. There was no effect of Nutri-Score C in combination with the respective claims (less sweet and 30% less sugar). The results are shown in Table 3.

**Table 3:** Frequencies of the health evaluation of three products with/without claim and Nutri-Score.

**Instant Cappuccino**	**Without claim**	**Less sweet**	**Without claim + Nutri-Score D**	**Less sweet + Nutri-Score D**	**Less sweet + Nutri-Score C**
**n**	232	216	210	233	209
**Healthy (%)**	30.2	41.2	17.6	30.9	43.1
**Unhealthy (%)**	69.8	58.8	82.4	69.4	56.9
**Oat drink**	**Without claim**	**Without added sugar**	**Without claim + Nutri-Score B**	**Without added sugar + Nutri-Score B**	**Without added sugar + Nutri-Score A**
**n**	212	239	230	182	240
**Healthy (%)**	84.0	84.5	91.3	87.9	90.0
**Unhealthy (%)**	16.0	15.5	8.7	12.1	10.0
**Chocolate crunchy muesli**	**Without claim**	**30% less sugar**	**Without claim + Nutri-Score D**	**30% less sugar + Nutri-Score D**	**30% less sugar + Nutri-Score C**
**n**	247	192	211	242	210
**Healthy (%)**	21.1	38.0	20.9	26.0	35.7
**Unhealthy (%)**	78.9	62.0	79.1	74.0	64.3

Note: Scale from: 1 = “very healthy” to 10 = “very unhealthy”. A rating of respondents was classified as healthy if the respondents rated it on the Likert scale with a value between 1–5, while a rating between 7–10 was classified as unhealthy.

The results of the ANOVA calculation illustrate product-specific differences in the effect on the various labelling variants (combinations of claims and Nutri-Score) on the health perception. In case of the chocolate crunchy muesli, the health assessment significantly increased when the sweet claim (30% less sugar) was present compared to the product without the sweet claim. Therefore, the health-halo effect can be confirmed for products with the worst health image at baseline. In the case of products on which the Nutri-Score was also displayed, it was shown that this had an influence on consumers. The Nutri-Score was able to correct the health image for products with a sweet or taste claim. This could be shown for some product variants for all three products regardless of whether they were initially assessed as healthy or unhealthy. For the rather unhealthy products (instant cappuccino and chocolate crunchy muesli), the taste claim (less sweet) and the sweet claim (30% less sugar) both increased the health assessment of the respective product, while the Nutri-Score C or Nutri-Score D were able to correct the health assessment. With the product perceived as healthy (oat drink), the nutrition claim (without added sugar) did not increase consumers’ health assessment of the product, while the Nutri-Score A increased the health assessment of the oat drink. Results can be found in Table 4.

**Table 4:** ANOVA of the health evaluation of three products with/without claim and Nutri-Score.

**Instant Cappuccino**	**Without claim**	**Less sweet**	**Without claim + Nutri-Score D**	**Less sweet + Nutri-Score D**	**Less sweet + Nutri-Score C**
**n**	232	216	210	233	209
**Mean**	6.63 ^a^	6.40 ^ac^	7.36 ^b^	6.73 ^a^	6.13 ^c^
**SD**	1.95	2.03	1.86	1.94	1.69
**Oat drink**	**Without claim**	**Without added sugar**	**Without claim + Nutri-Score B**	**Without added sugar + Nutri-Score B**	**Without added sugar + Nutri-Score A**
**n**	212	239	230	182	240
**Mean**	3.84 ^a^	3.89 ^a^	3.48 ^a^	3.75 ^a^	2.85 ^b^
**SD**	2.11	1.98	1.71	1.89	1.91
**Chocolate crunchy muesli**	**Without claim**	**30% less sugar**	**Without claim + Nutri-Score D**	**30% less sugar + Nutri-Score D**	**30% less sugar + Nutri-Score C**
**n**	247	192	211	242	210
**Mean**	7.13 ^a^	6.31 ^b^	7.24 ^a^	6.81 ^ac^	6.38 ^bc^
**SD**	1.86	1.98	1.99	2.01	1.78

Note: n = number of respondents, different letters a, b, indicate a significant (p < 0.05) difference between claims according to Games–Howell or Tukey Post hoc test, scale from: 1 = “very healthy” to 10 = “very unhealthy”.

## Discussion

Our results show that, when shopping for food, consumers pay most attention to the ingredients, followed by the sugar and the fat content of food items. This finding underlines the importance of the sugar content as a purchasing argument for foods. It has been shown that nutrition or taste claims related to sugar on front-of-pack labels can increase the health perception of the product. The results suggest that products with a poor health image at baseline, in particular, could be enhanced by nutrition claims. In this research, it can be assumed that the taste claim ‘less sweet’ may be considered as problematic, as consumers associate the claim ‘less sweet’ with a reduced sugar content.

None of the claims considered in this research ensure that the products labeled with them have a favorable nutritional profile. Neither the legally regulated nutrition claims nor the unregulated taste claim say anything about the natural sugar content of a product. In this respect, the claims are a poor indicator of the nutritional quality of a product. Even products with a high sugar content can use claims for advertisement. This means that consumers consider the products to be healthier even though this is not the case. In case of the less health-promoting products examples (instant cappuccino and chocolate crunchy muesli) the sweet claim can reinforce the health-halo effect only for the chocolate crunchy muesli. Do nutritional or taste claims trigger a health-halo effect, the Nutri-Score is able to correct consumers’ health assessment of the food item. One possible explanation why only the chocolate crunchy muesli yielded significant results could be the high discrepancy between consumers’ personal health assessment of the product and the classification of the Nutri-Score. Moreover, consumers did not distinguish between the legally regulated nutrition claims (30% less sugar, without added sugar) and the unregulated taste claims (less sweet). As a consequence, both types of claims led to distortions in consumers’ health assessments.

Another challenge is that many food purchase decisions are made under time-pressure [41] and are often habitual. Consumers only look at nutrition and taste claims for a few seconds [42]. Therefore, comprehensive information processing is not possible [42]. Additionally, research shows that consumers have a rather unconscious perception of such claims, as they do not remember looking at the claims after purchase [43]. If claims are on the front-of-product packaging, then ingredients and nutritional values tend to be considered less frequently [18]. A literature review by Kaur et al. [44] showed that such health-related claims have a substantial effect on food choice. Sugar claims have the potential to make food appear healthier than the same product without a sugar claim [23, 44].

From a nutrition policy perspective, nutrition claims, such as those on sugar reduction in foods, are intended to contribute to a health-promoting diet. At the same time, they are suitable for concealing the nutritional quality of foods. This occurs because the nutrition claims focus on one aspect of the food and thus mask other nutritional characteristics. In order to prevent misinformation about the nutritional quality of foods, the European legislator has introduced the Health Claims Regulation (EU-VONr.1924/2006) and regulated sub-areas of health marketing. Nevertheless, not all claims in the market are covered by the regulation and used by companies for marketing purposes only. The present study results concur with studies demonstrating health-halo effects for advertising with nutritional claims, which may result in unhealthy food choices and even in an increased calorie intake [29, 45]. The findings point to the problems when the content of a single nutrient is advertised. An increase in health assessment rating of food products was demonstrated for both legally regulated sugar claims (without added sugar, 30% less sugar) and the unregulated taste claim (less sweet). In case of the taste claim, however, the results were not significant, possibly due to product-specific differences. Past research has shown that such claims influence products to be evaluated as healthy [18, 46–48]. Mediano Stoltze et al. [30] used breakfast cereals as a product example and found that warning labels help to diminish the health-halo effect caused by nutrition claims. The red scale of the Nutri-Score with the rating of products with poor nutritional quality (D and E) may also be understood by consumers as a warning label. Research shows that consumers judge food products to consist of lower nutritional value [49] or to be more hazardous [50] when food labels of red color are on the packaging. Therefore, it can be concluded that consumers are particularly cautious about the color red used in food labels. Thus, the result from Mediano Stoltze et al. [30] is similar with the findings of the current study. For the product examples chocolate crunchy muesli and instant cappuccino, which had a negative Nutri-Score D rating (red), a corrective effect on consumers’ health assessment was demonstrated. In contrast, a Nutri-Score C label (yellow) on the instant cappuccino showed no significant effect. This indicates that a negative rating (D) is comparable to a warning label, while a medium rating (C) is perceived as neutral. All in all, the findings show the good comprehensibility of color-coded label concepts.

Given the negative impact that nutrition and taste claims could have on consumers’ perceptions of the healthiness of products, their use should be restricted. Policymakers could prohibit the use as such. But such comprehensive advertising bans are hardly politically enforceable. A less intrusive approach to regulation would be to limit the use to food products with minimum requirements for nutritional quality. The results of this study confirm research that has identified a positive effect of interpretive labels for a reality-based assessment of nutritional quality by consumers. A meta-analysis showed that food labelling, especially interpretive labels (e.g., traffic-light labeling and Nutri-Score), are an effective approach to empower consumers to choose healthier food products [51]. Additionally, the Nutri-Score has a positive effect on products purchased with high nutritional quality, followed by a decreasing effect on the purchase of products with medium and low nutritional quality [36]. Our study results suggest that misestimation of healthiness caused by nutrition and taste claims are reduced by using the Nutri-Score. Therefore, it would help if the Nutri-Score was mandatory, at least for companies that use such nutrition or taste claims on their products. Against the background of the European debate on the introduction of a mandatory system for simplified nutrition labeling, this is perhaps the most promising approach for regulation.

One limitation of this research is that the Nutri-Score was not established in Germany at the time of the online survey. However, the majority of participants had an idea about the meaning of the Nutri-Score. Therefore, it would be interesting to strengthen the results of this study once the Nutri-Score is well established and on many different products in retail stores. Moreover, only a limited number of product variants could be investigated in this study. To the best of our knowledge, this is the first study to analyze the contribution of the Nutri-Score on the perception of nutrition and taste claims about sugar. The results show the importance and need to discuss and regulate the role of advertising claims on consumers’ food choices.

From a nutrition policy perspective, labeling is a popular tool to support consumer decision-making. However, in order to anchor a health-promoting diet more broadly in society, a mix of instruments with other measures (e.g. sugar taxes) is required [52, 53].

## Conclusions

The sugar content of foods is important to consumers when purchasing. Food products with a poor health image can be enhanced by nutrition or taste claims related to sugar on front-of-pack labels. The mandatory introduction of the Nutri-Score, at least as a prerequisite for health marketing, could help to reduce problematic health effects caused by misleading claims. Compared to other instruments such as advertising bans or taxes, such a form of regulation would presumably be easier to implement.

Further investigation of the corrective effect of health-halo effects by the Nutri-Score should be explored for additional product categories, different Nutri-Score classifications, and additional advertising claims.
